# How different is the Learning Environment of Public and Private Sector Medical Colleges in Lahore, Pakistan?

**DOI:** 10.12669/pjms.39.3.6202

**Published:** 2023

**Authors:** Saadia Shahzad, Gohar Wajid

**Affiliations:** 1 Saadia Shahzad, M. Phil (Community Medicine) Associate Professor, Dept. of Community Medicine, Shalamar Medical and Dental College, Lahore, Pakistan; 2 Gohar Wajid, PhD (Medical Education), Health Professions Education Consultant, Pakistan

**Keywords:** JHLES, Learning environment, Medical colleges, Pakistan, Undergraduate, Students

## Abstract

**Background &Objective:**

Regular assessment of the Learning Environment (LE) of health professions education institutions is important for their continuous improvement and to keep the students motivated. Pakistan Medical & Dental Council (PM&DC) applies uniform standards of quality in all public and private sector medical colleges of the country. However, the learning environment of these colleges might be different due to differences in their geographic location, structure, utilization of resources, and modus operandi. This study was conducted to measure the learning environment in selected public and private sector medical colleges in the city of Lahore, Pakistan, using a pre validated instrument (Jhon Hopkins Learning Environment Scale).

**Methods:**

This cross-sectional descriptive study was conducted on 3,400 medical students from six public and private sector medical colleges of Lahore, during November and December 2020. Data was collected through Google forms. Two stage cluster random sampling technique was used to draw the study sample. John Hopkins Learning Environment Scale (JHLES) was used for data collection.

**Results:**

Overall JHLES mean score was 81.75 ±13.5. Public sector colleges had a significantly higher mean JHLES score (82.1) than private-sector colleges (81.1), with small effect size (0.083). Male students rated LE slightly higher than females (82.0 and 81.6 respectively).

**Conclusion:**

JHLES a relatively simpler tool (28 items) than DREEM, can be used effectively in the context of Pakistani environment to measure the LE in medical colleges. Both, public and private sector colleges had high overall JHLES mean scores, with public sector colleges having a significantly higher score than private-sector colleges.

## INTRODUCTION

Learning environment (LE), also called educational environment, encompasses perceptions of students about physical, social, and psychological learning contexts.^1^ In education, the role of the environment in motivation is of utmost importance. Arguably, education is all about designing learning environments that promote students’ motivation and learning.^2^ Institutions thus need to measure LE periodically, identify gaps and develop strategies to improve it. Understanding medical students’ perceptions of LE helps in effective planning and implementation of the curriculum. The way students perceive their LE is influenced by many factors like cultural diversity, available educational facilities, professional level of faculty, type of curriculum, and students’ expectations from the institution.^3^ World Federation for Medical Education (WFME) emphasizes that the educational environment should be addressed during the evaluation of educational programs.^4^

Measuring LE of a medical school is a complex task. No gold standard exists for assessing medical students’ perceptions of the LE. Measurement tools have evolved over time. Developing such a tool is a time consuming and resource intensive process. A close to ideal tool would possess strong validity evidence for content, response process, internal structure, and relationship to other variables. It would be efficient to administer, quick for participants to complete, widely applicable, and sensitive to change over time.^5^ Roff and colleagues developed Dundee Ready Education Environment Measure (DREEM) in 1997.^6^ It was reported as the most suitable tool to measure LE, used in over 40 studies from 20 different countries.^7^ In 2015, Shochet and colleagues developed a new tool named John Hopkins Learning Environment Scale (JHLES), claiming that current LE measuring instruments are not robust enough to assess the richness of the educational climate, at least in the USA.^1^

In Pakistan, several institutions have used DREEM to measure LE, we wanted to see how JHLES, as a new instrument would measure LE in the public and private sector medical colleges in Pakistan. JHLES is a relatively shorter (28 items divided into seven domains) instrument compared with DREEM (50 items divided into five domains), making it easy for students to respond. In addition to the measurement of the overall learning environment in public and private sector medical colleges, we also wanted to see if male students perceive learning environment differently than females.

## METHODS

It was a quantitative cross-sectional, descriptive study, conducted in six medical colleges from the city of Lahore, Pakistan. The target population for the study included all undergraduate medical students currently enrolled in public and private medical colleges in large cities of Pakistan. The accessible population included medical students from the colleges in the city of Lahore. It was a two-stage cluster random sample. All public and private sector medical colleges located in the city of Lahore were included in the study. In the first stage, three colleges from the private sector (Shalamar Medical and Dental College, CMH Lahore Medical College, University College of Medicine and Dentistry) and another three from the public sector (Allama Iqbal Medical College, Fatima Jinnah Medical University, and King Edward Medical University) were selected randomly.

Each college was taken as the main cluster and the five classes as mini clusters to ensure enough student representation from each class (see Table-I for overall class distribution). A total of 6750 students from the selected medical colleges formed the sampling frame of the study. A sample size of (3375, 50%); rounded off to 3400 was taken as acceptable. Every second student from the list of classes was included in the study. In case of non-response, the next student in the list was contacted to maintain the response rate. Ethical approval of the study was taken from the Research Ethics Committee at the University College of Medicine and Dentistry in November 2020 (approval number ERC/07/20/11).

**Table-I:** Demographic distribution of the study sample (N=3400).

Variable	N (%)
***Gender***	
Males	1008 (29.6)
Females	2392 (70.4)
***College type***	
Public	2117 (62.3)
Private	1283 (37.7)
***Age***	
≤ 22-year-old	2410 (70.9)
>22-year-old	990 (29.1)
***Class wise distribution***	
1^st^year	674 (19.8)
2^nd^year	637 (18.7)
3^rd^year	704 (20.7)
4^th^year	881(25.9)
Final year	504 (14.8)

John Hopkins Learning Environment Scale (JHLES) was used to measure the learning environment of the colleges. It has 28 items, distributed in seven domains, with an overall score of 28-140. The seven domains include the community of peers, faculty relationships, academic climate, meaningful engagement, mentoring, inclusion and safety, and physical space. Shochet and colleagues provide validity evidence of the tool in terms of its content, response process, internal structure, and relationship to other variables; for the local population of United States, where the study was conducted.^1^,^8^ The JHLES has been translated, adapted, assessed its psychometric properties, and used in several countries, including Brazil, Taiwan, India, China, Israel, and Malaysia. For convergent validity evidence of JHLES, Shochet and colleagues asked the students to complete a Personal Growth survey too, which had been developed by Wright and colleagues.^9^ At the local level, JHLES was shared with six medical educationists to find out its clarity of language and ease of reading. Although the questions were labelled as easy to understand, it was advised to replace the term ‘School of Medicine (SOM)’ with ‘medical college’ to align the questionnaire with Pakistani context. Confounding factors were considered at the data analysis stage and a homogenous study population i.e., undergraduate medical students from both sector colleges, both genders, and all five classes were recruited.

In our study, demographic variables of students included gender, age, class, and college type (public/private). The instrument was pilot tested on 30 students from two medical colleges, 15 each from public/private sectors to test its clarity and reading ease) in the local environment.

Data was collected during November and December 2020 through Google forms. Teachers and student representatives were actively involved to gain access to the students and maximize the response rate. The external validity was maintained through random selection of colleges from the sample frame in the first stage, applying cluster random sampling at the second stage, taking adequate sample size and maintaining rigorous study design. Standardized pre-validated questionnaire and data collection method were used to assure the quality of the study. Students were sensitized about the significance of the study repeatedly to take care of novelty and subject effects. Data analysis plan included checking the normality of the data and determining the reliability of the JHLES tool by Cronbach alpha.

## RESULTS

Our final sample included 1008 (29.6%) male and 2392 (70.4%) female students from the six colleges. Representation from both sector colleges was proportionate to the number of seats in these colleges. Table-I provides the distribution of demographics of students.

**Fig.1:**
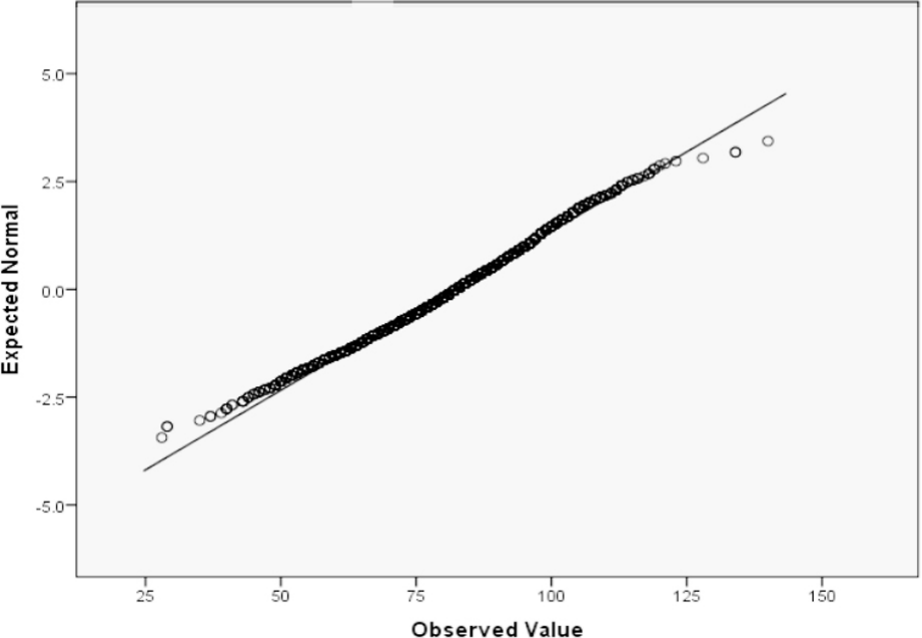
Frequency distribution of JHLES scores on Q-Q Plot (N=3400).

**Fig.2:**
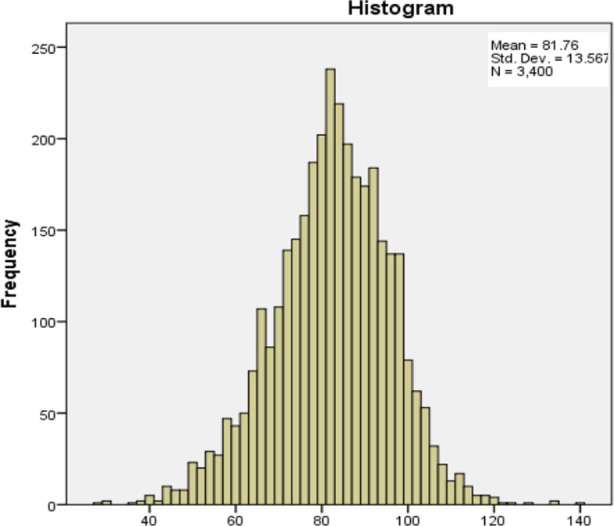
Frequency distribution of JHLES scores (N=3400).

JHLES showed strong internal consistency in our study (Cronbach’s Alpha = .88). Data from 3400 respondents showed Normal distribution on the Q-Q plot and Histogram, as evident from Fig.1 and 2. Table-II shows the distribution of the overall JHLES score with its seven domains. Scores for each question range on a five-point Likert Scale, with one as the lowest score and five as the highest score.

**Table-II:** Distribution of overall scores for JHLES (N=3400)

JHLES domains and number of questions	Range of score for the questions	Mean	SD
Overall JHLES (28 questions)	28-140	81.7	13.5
Community of peers (6)	6 – 30	17.4	4.0
Faculty relationships (6)	6 – 30	17.5	4.7
Academic climate (5)	5 – 25	15.2	3.5
Meaningful engagement (4)	4 – 20	11.1	3.0
Mentoring (2)	2 – 10	5.4	1.8
Inclusion and safety (3)	3 – 15	8.1	2.4
Physical space (2)	2 – 10	6.7	1.3

Results for the overall global perception of students about LE showed: 89 (2.6%) as terrible; 441 (13%) as poor; 1603 (47.1%) as fair; 1189 (35%) as good; and 78 (2.3%) as exceptional. Mean JHLES scores for public and private medical colleges were computed using Independent Sample *t*-test and 2-tailed significance. There was a significant difference in the overall score of LE of public and private sector colleges and all domains except ‘mentoring and physical space’. Effect size was however small for all domains except ‘meaningful engagement’. Table-III provides the distribution of results.

**Table-III:** Comparison of overall JHLES mean/domain scores between public and private sector medical colleges (N=3400).

Variable	Group	N	Mean Score	SD	p-value	Cohen’s d-Effect Sizes
JHLES mean score	Public	2117	82.1	13.4	.034*	.083
	Private	1283	81.1	13.7		
Community of peers	Public	2117	17.6	4.0	.009*	.090
	Private	1283	17.2	4.0		
Faculty relationships	Public	2117	17.4	4.7	.007*	-.082
	Private	1283	17.8	4.7		
Academic climate	Public	2117	15.3	3.4	.036*	.084
	Private	1283	15.1	3.7		
Meaningful engagement	Public	2117	11.6	2.9	.01*	.391
	Private	1283	10.4	3.1		
Mentoring	Public	2117	5.4	1.8	.847	.006
	Private	1283	5.4	1.8		
Inclusion and safety	Public	2117	8.0	2.3	.01*	-.153
	Private	1283	8.3	2.5		
Physical space	Public	2117	6.7	1.3	.533	.031
	Private	1283	6.7	1.3		

* p-value <.05 is taken significant.

Mean JHLES scores for males and females were computed using an Independent Sample *t*-test and 2-tailed significance. Significantly higher mean scores for males were noted in only two domains out of seven (mentoring and inclusion and safety). The distribution of JHLES scores for males and females is shown in Table-IV.

**Table-IV:** Distribution of JHLES scores for males and females (N=3400).

Variable	Groups	N	Mean	SD	p-value
Overall JHLES scores	Male	1008	82.0	14.2	.479
	Female	2392	81.6	13.2	
Community of peers	Male	1008	17.5	4.2	.286
	Female	2392	17.4	3.9	
Faculty relationships	Male	1008	17.4	4.9	.420
	Female	2392	17.6	4.6	
Academic climate	Male	1008	15.2	3.7	.461
	Female	2392	15.3	3.4	
Meaningful engagement	Male	1008	10.9	3.2	.456
	Female	2392	11.2	3.0	
Mentoring	Male	1008	5.5	1.9	.040*
	Female	2392	5.3	1.8	
Inclusion and safety	Male	1008	8.6	2.6	.01*
	Female	2392	7.9	2.3	
Physical space	Male	1008	6.7	1.4	.831
	Female	2392	6.7	1.3	

* p-value <.05 is taken significantly.

JHLES scores were compared for males and females at the level of public/private sector medical colleges. There is a possibility of observing a difference in perceptions of male and female students as we find a marked contextual difference in the environment of both sector colleges. Results showed a significant difference in the domains of ‘faculty relationship, mentoring and inclusion and safety in public colleges’, and for ‘meaningful engagement and inclusion and safety’ in the private sector. Table-V shows the distribution of results.

**Table-V:** Gender-based comparative scores at public/ private medical college level (N=3400).

College type	Variable	Groups	N	Mean	SD	p-value
Public	Overall JHLES score	Male	570	82.3	14.5	.741
		Female	1547	82.0	13.0	
	Community of peers	Male	570	17.7	4.3	.333
		Female	1547	17.5	3.9	
	Faculty relationships	Male	570	17.0	4.9	.030*
		Female	1547	17.5	4.6	
	Academic climate	Male	570	15.3	3.7	.679
		Female	1547	15.3	3.3	
	Meaningful engagement	Male	570	11.4	3.0	.129
		Female	1547	11.6	2.8	
	Mentoring	Male	570	5.5	1.9	.040*
		Female	1547	5.3	1.8	
	Inclusion and safety	Male	570	8.5	2.5	.01*
		Female	1547	7.8	2.2	
	Physical space	Male	570	6.7	1.5	.500
		Female	1547	6.7	1.3	
Private	Overall JHLES scores	Male	438	81.6	13.9	.340
		Female	854	80.8	13.6	
	Community of peers	Male	438	17.3	4.2	.411
		Female	854	17.1	3.9	
	Faculty relationships	Male	438	18.0	4.9	.307
		Female	854	17.7	4.6	
	Academic climate	Male	438	15.0	3.7	.686
		Female	854	15.1	3.6	
	Meaningful engagement	Male	438	10.2	3.3	.044*
		Female	854	10.5	3.1	
	Mentoring	Male	438	5.5	1.9	.162
		Female	854	5.3	1.8	
	Inclusion and safety	Male	438	8.7	2.6	.01*
		Female	854	8.1	2.4	
	Physical space	Male	438	6.7	1.3	.575
		Female	854	6.7	1.3	

* p-value <.05 is taken significantly.

## DISCUSSION

Favorable LE in an educational institution is linked with enhancing academic achievement, empathy, wellbeing, and reduce burnout and distress of students.^8^,^10^ Thus medical schools strive to provide a positive motivating learning environment to students. In the USA, Liaison Committee on Medial Education formally requires that a medical school ensures that medical programs occur in professional, respectful, and intellectually stimulating academic and clinical environments.^11^

JHLES showed high reliability in the Pakistani setup (Cronbach Alpha = .88), which is comparable with another study conducted in Malaysia (Cronbach Alpha = 0.92).^12^ High reliability and validity of the instrument and its domains were also observed in another validation study conducted in Brazil.^13^ Our study showed a Normal distribution pattern (Fig.1, 2), consistent with studies conducted in Malaysia (n = 367 students) and the USA (n = 377).^1^,^8^ Large sample size is a major strength of our study.

In this study, overall JHLES and its domains show high mean scores (Table-II). The study conducted in Malaysia reported higher overall JHLES mean scores and domain scores than the present study.^12^ Our study finding is consistent with a study conducted in two public sector medical colleges in India and higher trends of overall and domain mean scores were seen with a significant p-value for three out of seven domains.^14^ Our findings are also consistent with the results of the original study conducted by Shochet for the validation of JHLES and with the validation study conducted in Brazil.^13^ Study conducted by Zalts et al. on 622 medical students from three medical schools in Israel, Malaysia and China showed an overall mean 100.3 (SD=15.2).^15^ In another study conducted in USA, Dyrbye et al. used the six questions from ‘faculty relationships’ domain to compare the association between faculty relationships, and resident burnout. Mean score for the domain was 24.9, with Cronbach alpha for the JHLES-faculty relationship subscale was 0.93.^16^

Unlike DREEM, the developers of JHLES distribute the scores in high or low categories. Researchers can develop their own criteria as all parameters are measured on five-point Likert Scale. Scores may be divided into five equal categories with increment of 20%. Up to 28, may be rated as terrible, 29 to 56 = poor, 57 to 84 = fair, 85 to 112 = good, and 113 to 140 = exceptional. Zhou et al. conducted a large-scale study in 11 Chinese universities. They used cutoff points as high score (≥104) and low score as <104). In their study 5760 students (54.5%) ranked LE as high and 4816 (45.5%) ranked it as low.^17^

Our study shows an overall mean score of 81.7 (rated as fair), which is almost 59% of the overall score. Domain scores can also be divided, based on the above 20% increment rule. Results of students’ overall global perception of LE in the present study are compared with the Malaysian study that reported most students (>80%) had rated LE as exceptional.^10^A similar finding is there in the original study that stated higher mean scores of overall global perceptions for the fair, good, exceptional categories; and few for terrible/poor category. Thus, in the present study authors can safely state that the JHLES response process and content have shown consistency across different cultures.

In the present study, JHLES scores show a significantly higher mean for public sector medical colleges in all except two domains of ‘mentoring’ and ‘physical space’ (Table-III). This finding is similar to the Malaysian study, found higher overall JHLES mean scores for all the medical schools; along with significant p-value in ‘faculty relations, mentoring, safety and inclusion, and physical space’.^10^ Our study findings are also consistent with another study conducted in two public sector medical schools in India. The authors of Indian study found overall JHLES mean scores of 86.2±14.94 and 82.86±16.77. Mean scores for different domains showed a similar pattern as in this study and a significant p-value for ‘inclusion and safety/meaningful engagement/physical space’ domains.^12^ The study conducted by Tackett in Malaysia had students from five different ethnic groups who were taught a single curriculum adopted from JHUSOM. This same study also found a significant difference (p<.05) for the overall JHLES mean score and for five out of seven domains.^13^,^18^ In our study, Cohen’s d-effect size for public/ private mean JHLES scores is <.2, indicating the actual difference between the two groups is negligible, although statistically significant. Similarly, <.2 effect size for all the domains of JHLES except ‘meaningful engagement’ (.391) shows negligible mean difference for all domains, except for ‘meaningful engagement’.

In our study, males rated LE higher than females (Table-IV). A study conducted in Brazil on 248 medical students reported a higher mean score of 91.7 for males and 89.7 for females.^13^ Trends for the domain mean scores in our study are consistent with the study by Shochet that also shows higher mean domains scores for females in the ‘community of peers, faculty relations, academic climate, and physical space’; and significant p-value for ‘inclusion and safety’.^1^

In the present study, a gender-based comparison of scores at the two sectors level (Table-V) showed higher mean scores for males in overall JHLES scores, ‘the community of peers, mentoring, inclusion and safety’ in public medical colleges. The difference between domains ‘faculty relations, mentoring/ inclusion and safety’ was statistically significant. Whereas in private medical colleges, higher mean scores for males were noted in overall JHLES scores, ‘the community of peers, faculty relations, mentoring, inclusion and safety’; with a significant p-value for ‘meaningful engagement/inclusion and safety’. JHLES has picked up differences in four out of seven domains in both sector colleges as well as among the two genders. The areas identified with significant differences in this study need to be investigated in-depth for further improvement. Further studies are needed to identify the factors influencing the nature of the differences, to develop strategies to improve the LE.

Literature suggests that educational environment deeply relates to resident well-being and helps in understanding factors leading to student burnout and depression.^19^-^21^ Our study shows that males may perceive LE differently than females and it may be different for students in public and private sector institutions. Dyrbye et al. suggest that educational leaders should select and develop faculty who support, inspire, and connect well with students. They identify specific areas where faculty development may be most useful, including time management, learning climate, feedback, role-modeling life-long learning and self-care, and facilitating open dialog.

### Strengths of the study:

We conducted this study on a large sample of medical students (n=3400) in the six selected medical colleges in one metropolitan city, using probability sampling for representativeness and generalizability. This study adds new, extensive, and comparative knowledge about LE in our set up, using a new pre-validated instrument.

### Limitations of the study:

Getting access to the students, especially from other cities of the country was a major limitation, due to COVID-19 pandemic.

## CONCLUSION

The study shows high mean JHLES scores in both sectors medical colleges. At the public/private level high mean JHLES score is observed for the public medical colleges LE and a significant difference is noted in five out of the seven domains. Males perceive their LE as more positive and have rated it higher than females.

## References

[1] Shochet RB, Colbert-Getz JM, Wright SM (2015). The Johns Hopkins Learning vironment Scale:Measuring Medical Students'perceptions of the Processes Supporting Professional Formation. Acad Med.

[2] Kaplan A, Patrick H, Wentzel K, Miele D (2016). Learning environments and motivation. Handbook of motivation at school.

[3] Genn JM (2001). AMEE Medical Education Guide No.23 (Part 1):Curriculum, environment, climate, quality, and change in medical education- a unifying perspective. Med Teach.

[4] Riquelme A, Oporto M, Oporto J, Medez JI, Viviani P, Salech F (2009). Measuring students'perceptions of the educational climate of the New Curriculum at the Pontificia Universidad Catolica de Chile:performance of the Spanish translation of the Dundee Ready Educational Environment Measure DREEM. Educ for Health.

[5] Colbert-Getz JM, Kim S, Goode VH, Shochet RB, Wright SM (2014). Assessing medical students'and residents'perceptions of the learning environment:exploring validity evidence for the interpretation of scores from existing tools. Acad Med.

[6] Roff S, McAleer S, Ifere OS, Bhattacharya S (2001). A global diagnostic tool for measuring educational environment:comparing Nigeria and Nepal. Med Teach.

[7] Miles S, Swift L, Leinster SJ (2012). The Dundee Ready Education Environment Measure (DREEM):A review of its adoption and use. Med Teach.

[8] Tackett S, Wright S, Lubin R, Li J, Pan H (2017). International study of medical school learning environments and their relationship with student well-being and empathy. Med Educ.

[9] Wright SM, Levine RB, Beasley B (2006). Personal growth and its correlates during residency training. Med Educ.

[10] Karani R (2015). Enhancing the medical school learning environment:a complex challenge. J Gen Intern Med.

[11] Liaison Committee on Medical Education Functions and Structure of a Medical School.

[12] Tackett S, Bakar HA, Shilkofski NA, Coady N, Rampal K, Wright S (2015). Profiling medical school learning environments in Malaysia:a validation study of the JHLES. J Educ Eval Health Prof.

[13] Damiano RF, Furtado A, N da Silva B, Ezequiel OS, Lucchetti ALG, Dilalla LF (2020). Measuring students'perceptions of the medical school environment:Translation, Transcultural adaptation, and validation of two instruments to the Brazilian Portuguese language. J Med Educ and Curricular Dev.

[14] Tackett S, Shochet R, Shilkofski NA, Colbert-Getz J, Rampal K, Bakar HA (2015). Learning environment assessment of a single curriculum being taught at two medical schools 10,000 miles apart. BMC Med Educ.

[15] Zalts R, Green N, Tackett S, Lubin R (2021). The association between medical students'motivation with learning environment, perceived academic rank, and burnout. Int J Med Educ.

[16] Dyrbye LN, Leep Hunderfund AN, Moeschler S, Vaa B, Dozois E, Winters RC, Satele D, West CP (2021). Residents'Perceptions of Faculty Behaviors and Resident Burnout:a Cross-Sectional Survey Study Across a Large Health Care Organization. J Gen Intern Med.

[17] Zhou Z, Huang R, Zhang G, Gong M, Xian S, Yin H (2022). Nomograms for Predicting Medical Students'Perceptions of the Learning Environment:Multicenter Evidence from Medical Schools in China. Front Public Health.

[18] Sengupta P, Sharma A, Das N (2017). Perceptions of learning environment among undergraduate medical students in two different medical schools through DREEM and JHLES Questionnaires. J Clin and Diag Res.

[19] Dyrbye LN, Shanafelt TD (2016). A narrative review on burnout experienced by medical students and residents. Med Educ.

[20] Pereira-Lima K, Gupta RR, Guille C, Sen S (2019). Residency program factors associated with depressive symptoms in internal medicine interns:a prospective cohort study. Acad Med.

[21] Wasson LT, Cusmano A, Meli L, Louh I, Falzon L, Hampsey M (2016). Association between learning environment interventions and medical student well-being:a systematic review. JAMA.

